# A forward–backward penalty scheme with inertial effects for monotone inclusions. Applications to convex bilevel programming

**DOI:** 10.1080/02331934.2018.1556662

**Published:** 2018-12-11

**Authors:** Radu Ioan Boţ, Dang-Khoa Nguyen

**Affiliations:** a Research Platform Data Science @ Uni Vienna and Faculty of Mathematics, University of Vienna, Vienna Austria; b Faculty of Mathematics, University of Vienna, Vienna, Austria

**Keywords:** Maximally monotone operator, Fitzpatrick function, forward–backward splitting algorithm, convex bilevel optimization, 47H05, 65K05, 90C25

## Abstract

We investigate a forward–backward splitting algorithm of penalty type with inertial effects for finding the zeros of the sum of a maximally monotone operator and a cocoercive one and the convex normal cone to the set of zeroes of an another cocoercive operator. Weak ergodic convergence is obtained for the iterates, provided that a condition expressed via the Fitzpatrick function of the operator describing the underlying set of the normal cone is verified. Under strong monotonicity assumptions, strong convergence for the sequence of generated iterates is proved. As a particular instance we consider a convex bilevel minimization problem including the sum of a non-smooth and a smooth function in the upper level and another smooth function in the lower level. We show that in this context weak non-ergodic and strong convergence can be also achieved under inf-compactness assumptions for the involved functions.

## 1. Introduction and preliminaries

### 1.1. Motivation and problems formulation

During the last couple years one can observe in the optimization community an increasing interest in numerical schemes for solving variational inequalities expressed as monotone inclusion problems of the form

(1) $$0\in Ax+N_{M}\left(x\right),$$

where H is a real Hilbert space, A:H⇉H is a maximally monotone operator, M:=arg⁡minh is the set of global minima of the proper, convex and lower semicontinuous function $h:R\to \bar{R}:=R\cup \{\pm \infty \}$ and $N_{M}:H⇉H$ is the normal cone of the set *M*. The article [1] was starting point for a series of papers [2–12] addressing this topic or related ones. All these papers share the common feature that the proposed iterative schemes use penalization strategies, namely, they evaluate the penalized *h* by its gradient, in case the function is smooth (see, for instance, [3]), and by its proximal operator, in case it is non-smooth (see, for instance, [4]).

Weak ergodic convergence has been obtained in [3,4] under the hypothesis:

(2) $$\mathrm{For}\;\mathrm{all}\;p\in \mathrm{Ran}N_{M},\sum_{n\geq 1}\lambda_{n}\beta_{n}\left[h^{*}\left(\frac{p}{\beta_{n}}\right)-\sigma_{M}\left(\frac{p}{\beta_{n}}\right)\right]<+\infty ,$$

with $(\lambda_{n}{)}_{n\geq 1}$, the sequence of step sizes, $(\beta_{n}{)}_{n\geq 1}$, the sequence of penalty parameters, $h^{*}:H\to \bar{R}$, the Fenchel conjugate function of *h*, and $\mathrm{Ran}N_{M}$ the range of the normal cone operator $N_{M}:H⇉H$. Let us mention that (2) is the discretized counterpart of a condition introduced in [1] for continuous-time non-autonomous differential inclusions.

One motivation for studying numerical algorithms for monotone inclusions of type (1) comes from the fact that, when A≡∂f is the convex subdifferential of a proper, convex and lower semicontinuous function $f:H\to \bar{R}$, they furnish iterative methods for solving bilevel optimization problems of the form

(3) $$\min_{x\in H}\left\{f\left(x\right):x\in \arg\min h\right\}.$$

Among the applications where bilevel programming problems play an important role we mention the modelling of Stackelberg games, the determination of Wardrop equilibria for network flows, convex feasibility problems [13], domain decomposition methods for PDEs [14], image processing problems [6], and optimal control problems [4].

Later on, in [7], the following monotone inclusion problem, which turned out to be more suitable for applications, has been addressed in the same spirit of penalty algorithms

(4) $$0\in Ax+Dx+N_{M}\left(x\right),$$

where A:H⇉H is a maximally monotone operator, D:H→H is cocoercive operator and the constraint set *M* is the set of zeros of another cocoercive operator B:H→H. The provided algorithm of forward–backward type evaluates the operator *A* by a backward step and the two single-valued operators by forward steps. For the convergence analysis, (2) has been replaced by a condition formulated in terms of the Fitzpatrick function associated with the operator *B*, which we will also use in this paper. In [5], several particular situations for which this new condition is fulfilled have been provided.

The aim of this work is to endow the forward–backward penalty scheme for solving (4) from [7] with inertial effects, which means that the new iterate is defined in terms of the previous two iterates. Inertial algorithms have their roots in the time discretization of second-order differential systems [15]. They can accelerate the convergence of iterates when minimizing a differentiable function [16] and the convergence of the objective function values when minimizing the sum of a convex non-smooth and a convex smooth function [17,18]. Moreover, as emphasized in [19], see also [20], algorithms with inertial effects may detect optimal solutions of minimization problems which cannot be found by their non-inertial variants. In the last years, a huge interest in inertial algorithms can be noticed (see, for instance, [8,9,15,17,20–32]).

We prove weak ergodic convergence of the sequence generated by the inertial forward–backward penalty algorithm to a solution of the monotone inclusion problem (4), under reasonable assumptions for the sequences of step sizes, penalty and inertial parameters. When the operator *A* is assumed to be strongly monotone, we also prove strong convergence of the generated iterates to the unique solution of (4).

In Section 3, we address the minimization of the sum of a convex non-smooth and a convex smooth function with respect to the set of minimizes of another convex and smooth function. Besides the convergence results obtained from the general case, we achieve weak non-ergodic and strong convergence statements under inf-compactness assumptions for the involved functions. The weak non-ergodic theorem is an useful alternative to the one in [9], where a similar statement has been obtained for the inertial forward–backward penalty algorithm with constant inertial parameter under assumptions which are quite complicated and hard to verify (see also [11,12]).

### 1.2. Notations and preliminaries

In this subsection we introduce some notions and basic results which we will use throughout this paper (see [33–35]). Let H be a real Hilbert space with *inner product* ⟨⋅,⋅⟩ and associated *norm* $∥\cdot ∥=\sqrt{\left\langle \cdot ,\cdot \right\rangle }$.

For a function $\Psi :H\to \bar{R}:=R\cup \{\pm \infty \}$, we denote DomΨ={x∈H:Ψ(x)<+∞} its *effective domain* and say that Ψ is *proper*, if DomΨ≠∅ and Ψ(x)>−∞ for all x∈H. The *conjugate function* of Ψ is $\Psi^{*}:H\to \bar{R},\Psi^{*}(u)=\underset{x\in H}{\sup}\{\langle x,u\rangle -\Psi (x)\}$. The *convex subdifferential* of Ψ at the point x∈H is the set ∂Ψ(x)={p∈H:⟨y−x,p⟩≤Ψ(y)−Ψ(x) ∀y∈H}, whenever Ψ(x)∈R. We take by convention ∂Ψ(x)=∅, if Ψ(x)∈{±∞}.

Let *M* be a non-empty subset of H. The *indicator function* of *M*, which is denoted by $\delta_{M}:H\to \bar{R}$, takes the value 0 on *M* and +∞ otherwise. The *convex subdifferential* of the *indicator function* is the *normal cone* of *M*, that is $N_{M}(x)=\{p\in H:\langle y-x,p\rangle \leq \;0\;\forall y\in H\}$, if x∈M, and $N_{M}(x)=\emptyset$ otherwise. Notice that for x∈M we have $p\in N_{M}(x)$ if and only if $\sigma_{M}(x)=\langle x,p\rangle$, where $\sigma_{M}=\delta_{M}^{*}$ is the *support function* of *M*.

For an arbitrary set-value operator A:H⇉H we denote by GrA={(x,v)∈H×H:v∈Ax} its *graph*, by DomA={x∈H:Ax≠∅} its *domain*, by RanA={v∈H:∃x∈H with v∈Ax} its *range* and by $A^{-1}:H⇉H$ its *inverse operator*, defined by $(v,x)\in \mathrm{Gr}A^{-1}$ if and only if (x,v)∈GrA. We use also the notation ZerA={x∈H:0∈Ax} for the *set of zeros* of the operator *A*. We say that *A* is *monotone*, if ⟨x−y,v−w⟩≥0 for all (x,v),(y,w)∈GrA. A monotone operator *A* is said to be *maximally monotone*, if there exists no proper monotone extension of the graph of *A* on H×H. Let us mention that if *A* is maximally monotone, then ZerA is a convex and closed set [33, Proposition 23.39]. We refer to [33, Section 23.4] for conditions ensuring that ZerA is non-empty. If *A* is maximally monotone, then one has the following characterization for the set of its zeros

(5) $$z\in \mathrm{Zer}\;A\;\mathrm{if}\;\mathrm{and}\;\mathrm{only}\;\mathrm{if}\;\left\langle u-z,y\right\rangle \geq 0\;\mathrm{for}\;\mathrm{all}\;\left(u,y\right)\in \mathrm{Gr}\;A.$$

The operator *A* is said to be *γ*- *strongly monotone* with γ>0, if $\langle x-y,v-w\rangle \geq ∥x-y{∥}^{2}$ for all (x,v),(y,w)∈GrA. If *A* is maximally monotone and strongly monotone, then ZerA is a singleton, thus non-empty [33, Corollary 23.27].

The *resolvent* of $A,J_{A}:H⇉H$, is defined by $J_{A}:=(\mathrm{Id}+A{)}^{-1}$, where Id:H→H denotes the *identity operator* on H. If *A* is maximally monotone, then $J_{A}:H\to H$ is single-value and maximally monotone [33, Proposition 23.7, Corollary 23.10]. For an arbitrary γ>0, we have the following identity [33, Proposition 23.18]

$$J_{\gamma A}+\gamma J_{\gamma^{-1}A^{-1}}\circ \gamma^{-1}\mathrm{Id}=\mathrm{Id}.$$

We denote Γ(H) the family of proper, convex and lower semicontinuous extended real-valued functions defined on H. When Ψ∈Γ(H) and γ>0, we denote by ${\mathrm{prox}}_{\gamma \Psi }(x)$ the *proximal point* with parameter *γ* of function Ψ at point x∈H, which is the unique optimal solution of the optimization problem

$$\underset{y\in H}{\inf}\left\{\Psi \left(y\right)+\frac{1}{2\gamma }{∥y-x∥}^{2}\right\}.$$

Notice that $J_{\gamma \partial \Psi }=(\mathrm{Id}+\gamma \partial \Psi {)}^{-1}={\mathrm{prox}}_{\gamma \Psi }$, thus ${\mathrm{prox}}_{\gamma \Psi }:H\to H$ is a single-valued operator fulfilling the so-called *Moreau's decomposition formula*:

$${\mathrm{prox}}_{\gamma \Psi }+\gamma {\mathrm{prox}}_{\gamma^{-1}\Psi^{*}}\circ \gamma^{-1}\mathrm{Id}=\mathrm{Id}.$$

The function $\Psi :H\to \bar{R}$ is said to be γ−*strongly convex* with γ>0, if $\Psi -\frac{\gamma }{2}∥\cdot {∥}^{2}$ is a convex function. This property implies that ∂Ψ is γ−strongly monotone.

The *Fitzpatrick function* [36] associated to a monotone operator *A* is defined as

$$\phi_{A}:H\times H\to \bar{R},\;\phi_{A}\left(x,u\right):=\underset{\left(y,v\right)\in \mathrm{Gr}A}{\sup}\left\{\left\langle x,v\right\rangle +\left\langle y,u\right\rangle -\left\langle y,v\right\rangle \right\}$$

and it is a convex and lower semicontinuous function. For insights in the outstanding role played by the Fitzpatrick function in relating the convex analysis with the theory of monotone operators we refer to [33,34,37–39] and the references therein. If *A* is maximally monotone, then $\phi_{A}$ is proper and it fulfills

$$\phi_{A}\left(x,u\right)\geq \left\langle x,u\right\rangle \;\forall \left(x,u\right)\in H\times H,$$

with equality if and only if (x,u)∈GrA. Notice that if Ψ∈Γ(H), then ∂Ψ is a maximally monotone operator and it holds $(\partial \Psi {)}^{-1}=\partial \Psi^{*}$. Furthermore, the following inequality is true (see [37]):

(6) $$\phi_{\partial \Psi }\left(x,u\right)\leq \Psi \left(x\right)+\Psi^{*}\left(u\right)\;\forall \left(x,v\right)\in H\times H.$$

We present as follows some statements that will be essential when carrying out the convergence analysis. Let $(x_{n}{)}_{n\geq 0}$ be a sequence in H and $(\lambda_{n}{)}_{n\geq 1}$ be a sequence of positive real numbers. The *sequence of weighted averages* $(z_{n}{)}_{n\geq 1}$ is defined for every n≥1 as

(7) $$z_{n}:=\frac{1}{\tau_{n}}\sum_{k=1}^{n}\lambda_{k}x_{k},\mathrm{where}\;\tau_{n}:=\sum_{k=1}^{n}\lambda_{k}.$$

Lemma 1.1 **Opial-Passty**

Let *Z* be a non-empty subset of H and assume that the limit $\lim_{n\to +\infty }∥x_{n}-u∥$ exists for every element u∈Z. If every sequential weak cluster point of $(x_{n}{)}_{n\geq 0},$ respectively $(z_{n}{)}_{n\geq 1},$ lies in *Z*, then the sequence $(x_{n}{)}_{n\geq 0},$ respectively $(z_{n}{)}_{n\geq 1},$ converges weakly to an element in *Z* as n→+∞.

Two following result can be found in [5,7].

Lemma 1.2 Let $(\theta_{n}{)}_{n\geq 0},(\xi_{n}{)}_{n\geq 1}$ and $(\delta_{n}{)}_{n\geq 1}$ be sequences in $R_{+}$ with $(\delta_{n}{)}_{n\geq 1}\in \ell^{1}$. If there exists $n_{0}\geq 1$ such that

$$\theta_{n+1}-\theta_{n}\leq \alpha_{n}\left(\theta_{n}-\theta_{n-1}\right)-\xi_{n}+\delta_{n}\;\forall n\geq n_{0}$$

and *α* such that

$$0\leq \alpha_{n}\leq \alpha <1\;\forall n\geq 1,$$

then the following statements are true:

- $\sum_{n\geq 1}[\theta_{n}-\theta_{n-1}{]}_{+}<+\infty$, where $\left[s{]}_{+}:=\max\{s,0\right\}$;
- the limit $\lim_{n\to \infty }\theta_{n}$ exists.
- the sequence $(\xi_{n}{)}_{n\geq 1}$ belongs to $\ell^{1}$.

The following result follows from Lemma 1.2, applied in case $\alpha_{n}:=0$ and $\theta_{n}:=\rho_{n}-\rho$ for all n≥1, where *ρ* is a lower bound for $(\rho_{n}{)}_{n\geq 1}$.

Lemma 1.3 Let $(\rho_{n}{)}_{n\geq 1}$ be a sequence in R, which is bounded from below, and $(\xi_{n}{)}_{n\geq 1}$, $(\delta_{n}{)}_{n\geq 1}$ be sequences in $R_{+}$ with $(\delta_{n}{)}_{n\geq 1}\in \ell^{1}$. If there exists $n_{0}\geq 1$ such that

$$\rho_{n+1}\leq \rho_{n}-\xi_{n}+\delta_{n}\;\forall n\geq n_{0},$$

then the following statements are true:

- the sequence $(\rho_{n}{)}_{n\geq 1}$ is convergent.
- the sequence $(\xi_{n}{)}_{n\geq 1}$ belongs to $\ell^{1}$.

The following result, which will be useful in this work, shows that statement (ii) in Lemma 1.3 can be obtained also when $(\rho_{n}{)}_{n\geq 1}$ is not bounded from below, but it has a particular form.

Lemma 1.4 Let $(\rho_{n}{)}_{n\geq 1}$ be a sequence in R and $(\xi_{n}{)}_{n\geq 1},(\delta_{n}{)}_{n\geq 1}$ be sequences in $R_{+}$ with $(\delta_{n}{)}_{n\geq 1}\in \ell^{1}$ and

$$\rho_{n}:=\theta_{n}-\alpha_{n}\theta_{n-1}+\chi_{n}\;\forall n\geq 1,$$

where $(\theta_{n}{)}_{n\geq 0},(\chi_{n}{)}_{n\geq 1}$ are sequences in $R_{+}$ and there exists *α* such that

$$0\leq \alpha_{n}\leq \alpha <1\;\forall n\geq 1.$$

If there exists $n_{0}\geq 1$ such that

(8) $$\rho_{n+1}-\rho_{n}\leq -\xi_{n}+\delta_{n}\;\forall n\geq n_{0},$$

then the sequence $(\xi_{n}{)}_{n\geq 1}$ belongs to $\ell^{1}$.

Proof. We fix an integer $\bar{N}\geq n_{0}$, sum up the inequalities in (8) for $n=n_{0},n_{0}+1,\ldots ,\bar{N}$ and obtain

(9) $$\rho_{\bar{N}+1}-\rho_{n_{0}}\leq -\sum_{n=n_{0}}^{\bar{N}}\xi_{n}+\sum_{n=n_{0}}^{\bar{N}}\delta_{n}\leq \sum_{n\geq 1}\delta_{n}<+\infty .$$

Hence the sequence $\{\rho_{n}{\}}_{n\geq 1}$ is bounded from above. Let $\bar{\rho }>0$ be an upper bound of this sequence. For all n≥1 it holds

$$\theta_{n}-\alpha \theta_{n-1}\leq \theta_{n}-\alpha_{n}\theta_{n-1}+\chi_{n}=\rho_{n}\leq \bar{\rho },$$

from which we deduce that

(10) $$-\rho_{n}\leq -\theta_{n}+\alpha \theta_{n-1}\leq \alpha \theta_{n-1}.$$

By induction we obtain for all $n\geq n_{0}+1$

(11) $$\theta_{n}\leq \alpha \theta_{n-1}+\bar{\rho }\leq \cdots \leq \alpha^{n-n_{0}}\theta_{n_{0}}+\bar{\rho }\sum_{k=1}^{n-n_{0}}\alpha^{k-1}\leq \alpha^{n-n_{0}}\theta_{n_{0}}+\frac{\bar{\rho }}{1-\alpha }.$$

Then inequality (9) combined with (10) and (11) leads to

(12) $$\begin{array}{rl} \sum_{n=n_{0}}^{\bar{N}}\xi_{n} & \leq \rho_{n_{0}}-\rho_{\bar{N}+1}+\sum_{n=n_{0}}^{\bar{N}}\delta_{n}\leq \rho_{n_{0}}+\alpha \theta_{\bar{N}}+\sum_{n\geq 1}\delta_{n}\\ & \leq \rho_{n_{0}}+\alpha^{\bar{N}-n_{0}+1}\theta_{n_{0}}+\frac{\alpha \bar{\rho }}{1-\alpha }+\sum_{n\geq 1}\delta_{n}<+\infty . \end{array}$$

We let $\bar{N}$ converge to +∞ and obtain that $\sum_{n\geq 1}\xi_{n}<+\infty$.

## 2. The general monotone inclusion problem

In this section we address the following monotone inclusion problem.

Problem 2.1 Let H be a real Hilbert space, A:H⇉H a maximally monotone operator, D:H→H an η−cocoercive with η>0,B:H→H a μ−cocoercive with μ>0 and assume that M:=Zer B≠∅. The monotone inclusion problem to solve reads

$$0\in Ax+Dx+N_{M}\left(x\right).$$

The following forward–backward penalty algorithm with inertial effects for solving Problem 2.1 will be in the focus of our investigations in this paper.


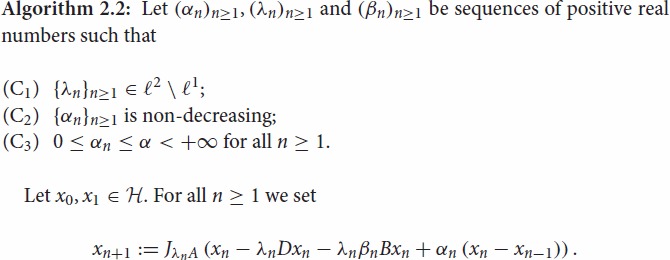


When *D*=0 and B=∇h, where h:H→R is a convex and differentiable function with $\mu^{-1}$-Lipschitz continuous gradient with μ>0 fulfilling minh=0, then Problem 2.1 recovers the monotone inclusion problem addressed in [3, Section 3] and Algorithm 2.2 can be seen as an inertial version of the iterative scheme considered in this paper. When *B*=0, we have that $N_{M}=\{0\}$ and Algorithm 2.2 is nothing else than the inertial version of the classical forward–backward algorithm (see for instance [33,40]).

Hypotheses 2.2 The convergence analysis will be carried out in the following hypotheses (see also [7]):

1. $A+N_{M}\text{is maximally monotone and }\mathrm{Zer}(A+D+N_{M})\neq \emptyset$;
2. for every $p\in \mathrm{Ran}N_{M},\sum_{n\geq 1}\lambda_{n}\beta_{n}[\underset{u\in M}{\sup}\phi_{B}(u,\frac{p}{\beta_{n}})-\sigma_{M}(\frac{p}{\beta_{n}})]<+\infty$.

Since *A* and $N_{M}$ are maximally monotone operators, the sum $A+N_{M}$ is maximally monotone, provided some specific regularity conditions are fulfilled (see [33–35,38]). Furthermore, since *D* is also maximally monotone [33, Example 20.28] and DomD≡H, if $A+N_{M}$ is maximally monotone, then $A+D+N_{M}$ is also maximally monotone.

Let us also notice that for $p\in \mathrm{Ran}N_{M}$ there exists $\hat{u}\in M$ such that $p\in N_{M}(\hat{u})$, hence, for every β>0 it holds

$$\underset{u\in M}{\sup}\phi_{B}\left(u,\frac{p}{\beta }\right)-\sigma_{M}\left(\frac{p}{\beta }\right)\geq \left\langle \hat{u},\frac{p}{\beta }\right\rangle -\sigma_{M}\left(\frac{p}{\beta }\right)=0.$$

For situations where ($H_{2}^{\mathrm{fitz}}$) is satisfied we refer the reader to [5,8,9,11].

Before formulating the main theorem of this section we will prove some useful technical results.

Lemma 2.3 Let $(x_{n}{)}_{n\geq 0}$ be the sequence generated by *Algorithm* 2.2 and (u,y) be an element in $\mathrm{Gr}(A+D+N_{M})$ such that *y*=*v*+*Du*+*p* with v∈Au and $p\in N_{M}(u)$. Further, let $\varepsilon_{1},\varepsilon_{2},\varepsilon_{3}>0$ be such that $1-\varepsilon_{3}>0$. Then the following inequality holds for all n≥1

(13) $$\begin{array}{rl} & {∥x_{n+1}-u∥}^{2}-{∥x_{n}-u∥}^{2}\\ \leq & \;\alpha_{n}{∥x_{n}-u∥}^{2}-\alpha_{n}{∥x_{n-1}-u∥}^{2}-\left(1-4\varepsilon_{1}-\varepsilon_{2}\right){∥x_{n+1}-x_{n}∥}^{2}\\ & \;+\left(\alpha_{n}+\frac{\alpha_{n}^{2}}{4\varepsilon_{1}}\right){∥x_{n}-x_{n-1}∥}^{2}+\left(\frac{2}{\varepsilon_{2}}\lambda_{n}^{2}\beta_{n}^{2}-2\mu \left(1-\varepsilon_{3}\right)\lambda_{n}\beta_{n}\right){∥Bx_{n}∥}^{2}\\ & \;+\left(\frac{4}{\varepsilon_{2}}\lambda_{n}^{2}-2\eta \lambda_{n}\right){∥Dx_{n}-Du∥}^{2}+\frac{4}{\varepsilon_{2}}\lambda_{n}^{2}{∥Du+v∥}^{2}\\ & \;+2\varepsilon_{3}\lambda_{n}\beta_{n}\left[\underset{u\in M}{\sup}\phi_{B}\left(u,\frac{p}{\varepsilon_{3}\beta_{n}}\right)-\sigma_{M}\left(\frac{p}{\varepsilon_{3}\beta_{n}}\right)\right]+2\lambda_{n}\left\langle u-x_{n},y\right\rangle . \end{array}$$

Proof. Let n≥1 be fixed. According to definition of the resolvent of the operator *A* we have

(14) $$x_{n}-x_{n+1}-\lambda_{n}\left(Dx_{n}+\beta_{n}Bx_{n}\right)+\alpha_{n}\left(x_{n}-x_{n-1}\right)\in \lambda_{n}Ax_{n+1}$$

and, since $\lambda_{n}v\in \lambda_{n}Au$, the monotonicity of *A* guarantees

(15) $$\left\langle x_{n+1}-u,x_{n}-x_{n+1}-\lambda_{n}\left(Dx_{n}+\beta_{n}Bx_{n}+v\right)+\alpha_{n}\left(x_{n}-x_{n-1}\right)\right\rangle \geq 0$$

or, equivalently,

(16) $$\begin{array}{rl} & 2\left\langle u-x_{n+1},x_{n}-x_{n+1}\right\rangle \leq 2\lambda_{n}\left\langle u-x_{n+1},\beta_{n}Bx_{n}+Dx_{n}+v\right\rangle \\ & \;-2\alpha_{n}\left\langle u-x_{n+1},x_{n}-x_{n-1}\right\rangle . \end{array}$$

For the term in the left-hand side of (16) we have

(17) $$2\left\langle u-x_{n+1},x_{n}-x_{n+1}\right\rangle ={∥x_{n+1}-u∥}^{2}+{∥x_{n+1}-x_{n}∥}^{2}-{∥x_{n}-u∥}^{2}.$$

Since

$$-2\alpha_{n}\left\langle u-x_{n},x_{n}-x_{n-1}\right\rangle \;=\;\;-\alpha_{n}{∥u\;-\;x_{n-1}∥}^{2}+\alpha_{n}{∥u\;-\;x_{n}∥}^{2}+\alpha_{n}{∥x_{n}\;-\;x_{n-1}∥}^{2}$$

and

$$2\left\langle x_{n+1}-x_{n},\alpha_{n}\left(x_{n}-x_{n-1}\right)\right\rangle \leq \;4\varepsilon_{1}{∥x_{n+1}-x_{n}∥}^{2}+\frac{\alpha_{n}^{2}}{4\varepsilon_{1}}{∥x_{n}-x_{n-1}∥}^{2},$$

by adding the two inequalities, we obtain the following estimation for the second term in the right-hand side of (16)

(18) $$\begin{array}{rl} & -2\alpha_{n}\left\langle u-x_{n+1},x_{n}-x_{n-1}\right\rangle \\ & \;\leq \;\alpha_{n}{∥x_{n}-u∥}^{2}-\alpha_{n}{∥x_{n-1}-u∥}^{2}+4\varepsilon_{1}{∥x_{n+1}-x_{n}∥}^{2}\\ & \;+\left(\alpha_{n}+\frac{\alpha_{n}^{2}}{4\varepsilon_{1}}\right){∥x_{n}-x_{n-1}∥}^{2}. \end{array}$$

We turn now our attention to the first term in the right-hand side of (16), which can be written as

(19) $$\begin{array}{rl} & 2\lambda_{n}\left\langle u-x_{n+1},\beta_{n}Bx_{n}+Dx_{n}+v\right\rangle \\ & \;=\;2\lambda_{n}\left\langle u-x_{n},\beta_{n}Bx_{n}+Dx_{n}+v\right\rangle +2\lambda_{n}\beta_{n}\left\langle x_{n}-x_{n+1},Bx_{n}\right\rangle \\ & \;+2\lambda_{n}\left\langle x_{n}-x_{n+1},Dx_{n}+v\right\rangle . \end{array}$$

We have

(20) $$2\lambda_{n}\beta_{n}\left\langle x_{n}-x_{n+1},Bx_{n}\right\rangle \leq \frac{\varepsilon_{2}}{2}{∥x_{n+1}-x_{n}∥}^{2}+\frac{2}{\varepsilon_{2}}\lambda_{n}^{2}\beta_{n}^{2}{∥Bx_{n}∥}^{2}$$

and

(21) $$\begin{array}{rl} 2\lambda_{n}\left\langle x_{n}-x_{n+1},Dx_{n}+v\right\rangle & \leq \frac{\varepsilon_{2}}{2}{∥x_{n+1}-x_{n}∥}^{2}+\frac{2}{\varepsilon_{2}}\lambda_{n}^{2}{∥Dx_{n}+v∥}^{2}\\ & \leq \;\frac{\varepsilon_{2}}{2}{∥x_{n+1}-x_{n}∥}^{2}+\frac{4}{\varepsilon_{2}}\lambda_{n}^{2}{∥Dx_{n}-Du∥}^{2}\\ & \;+\frac{4}{\varepsilon_{2}}\lambda_{n}^{2}{∥Du+v∥}^{2}. \end{array}$$

On the other hand, we have

(22) $$\begin{array}{rl} & 2\lambda_{n}\left\langle u-x_{n},\beta_{n}Bx_{n}+Dx_{n}+v\right\rangle \\ & \;=\;2\lambda_{n}\beta_{n}\left\langle u-x_{n},Bx_{n}\right\rangle +2\lambda_{n}\left\langle u-x_{n},Dx_{n}-Du\right\rangle +2\lambda_{n}\left\langle u-x_{n},Du+v\right\rangle . \end{array}$$

Since $0<\varepsilon_{3}<1$ and *Bu*=0, the cocoercivity of *B* gives us

(23) $$2\lambda_{n}\beta_{n}\left\langle u-x_{n},Bx_{n}\right\rangle \leq -2\mu \left(1-\varepsilon_{3}\right)\lambda_{n}\beta_{n}{∥Bx_{n}∥}^{2}+2\varepsilon_{3}\lambda_{n}\beta_{n}\left\langle u-x_{n},Bx_{n}\right\rangle .$$

Similarly, the cocoercivity of *D* gives us

(24) $$2\lambda_{n}\left\langle u-x_{n},Dx_{n}-Du\right\rangle \leq -2\eta \lambda_{n}{∥Dx_{n}-Du∥}^{2}.$$

Combining (23)–(24) with (22) and by using the definition Fitzpatrick function and the fact that $\sigma_{M}(p/\varepsilon_{3}\beta_{n})=\langle u,p/\varepsilon_{3}\beta_{n}\rangle$, we obtain

(25) $$\begin{array}{rl} & 2\lambda_{n}\left\langle u-x_{n},\beta_{n}Bx_{n}+Dx_{n}+v\right\rangle \\ & \;\leq -2\mu \left(1-\varepsilon_{3}\right)\lambda_{n}\beta_{n}{∥Bx_{n}∥}^{2}+2\varepsilon_{3}\lambda_{n}\beta_{n}\left\langle u-x_{n},Bx_{n}\right\rangle -2\eta \lambda_{n}{∥Dx_{n}-Du∥}^{2}\\ & \;+2\lambda_{n}\left\langle u-x_{n},Du+v\right\rangle \\ & \;=-2\mu \left(1-\varepsilon_{3}\right)\lambda_{n}\beta_{n}{∥Bx_{n}∥}^{2}+2\varepsilon_{3}\lambda_{n}\beta_{n}\left\langle u-x_{n},Bx_{n}\right\rangle -2\eta \lambda_{n}{∥Dx_{n}-Du∥}^{2}\\ & \;+2\lambda_{n}\left\langle u-x_{n},y-p\right\rangle \\ & \;=-2\mu \left(1-\varepsilon_{3}\right)\lambda_{n}\beta_{n}{∥Bx_{n}∥}^{2}-2\eta \lambda_{n}{∥Dx_{n}-Du∥}^{2}+2\lambda_{n}\left\langle u-x_{n},y\right\rangle \\ & \;+2\varepsilon_{3}\lambda_{n}\beta_{n}\left(\left\langle u,Bx_{n}\right\rangle +\left\langle x_{n},\frac{p}{\varepsilon_{3}\beta_{n}}\right\rangle -\left\langle x_{n},Bx_{n}\right\rangle -\left\langle u,\frac{p}{\varepsilon_{3}\beta_{n}}\right\rangle \right)\\ & \;\leq -2\mu \left(1-\varepsilon_{3}\right)\lambda_{n}\beta_{n}{∥Bx_{n}∥}^{2}-2\eta \lambda_{n}{∥Dx_{n}-Du∥}^{2}+2\lambda_{n}\left\langle u-x_{n},y\right\rangle \\ & \;+2\varepsilon_{3}\lambda_{n}\beta_{n}\left[\underset{u\in M}{\sup}\phi_{B}\left(u,\frac{p}{\varepsilon_{3}\beta_{n}}\right)-\sigma_{M}\left(\frac{p}{\varepsilon_{3}\beta_{n}}\right)\right]. \end{array}$$

The inequalities (20), (21) and (25) lead to

(26) $$\begin{array}{rl} & 2\lambda_{n}\left\langle u-x_{n+1},\beta_{n}Bx_{n}+Dx_{n}+v\right\rangle \\ & \;\leq \;\left(\frac{2}{\varepsilon_{2}}\lambda_{n}^{2}\beta_{n}^{2}-2\mu \left(1-\varepsilon_{3}\right)\lambda_{n}\beta_{n}\right){∥Bx_{n}∥}^{2}+\left(\frac{4}{\varepsilon_{2}}\lambda_{n}^{2}-2\eta \lambda_{n}\right)∥Dx_{n}\\ & \;{-Du∥}^{2}+\varepsilon_{2}{∥x_{n+1}-x_{n}∥}^{2}+\frac{4}{\varepsilon_{2}}\lambda_{n}^{2}{∥Du+v∥}^{2}\\ & \;+2\varepsilon_{3}\lambda_{n}\beta_{n}\left[\underset{u\in M}{\sup}\phi_{B}\left(u,\frac{p}{\varepsilon_{3}\beta_{n}}\right)-\sigma_{M}\left(\frac{p}{\varepsilon_{3}\beta_{n}}\right)\right]+2\lambda_{n}\left\langle u-x_{n},y\right\rangle . \end{array}$$

Finally, by combining (17), (18) and (26), we obtain (13).

From now on we will assume that for $0<\alpha <\frac{1}{3}$ the constants $\varepsilon_{1},\varepsilon_{2},\varepsilon_{3}>0$ and the sequences $(\lambda_{n}{)}_{n\geq 1}$ and $(\beta_{n}{)}_{n\geq 1}$ are chosen such that

$$(C_{4})\;\;1-\varepsilon_{3}>0,\;\;\varepsilon_{2}<1-4\varepsilon_{1}-\alpha -\frac{\alpha^{2}}{4\varepsilon_{1}}\;\;\mathrm{and}\;\;\underset{n\geq 1}{\sup}\lambda_{n}\beta_{n}<\mu \varepsilon_{2}\left(1-\varepsilon_{3}\right).$$

As a consequence, there exists $0<s\leq \;1-\frac{\varepsilon_{1}}{1-3\varepsilon_{1}-\varepsilon_{2}}{\left(1+\frac{\alpha }{2\varepsilon_{1}}\right)}^{2}$ , which means that for all n≥1 it holds

(27) $$\alpha_{n+1}+\frac{\alpha_{n+1}^{2}}{4\varepsilon_{1}}-\left(1-4\varepsilon_{1}-\varepsilon_{3}\right)\leq \alpha +\frac{\alpha^{2}}{4\varepsilon_{1}}-\left(1-4\varepsilon_{1}-\varepsilon_{3}\right)<-s.$$

On the other hand, there exists $0<t\leq \mu (1-\varepsilon_{2})-(1/\varepsilon_{3})\underset{n\geq 0}{\sup}\lambda_{n}\beta_{n}$ , which means that for all n≥1 it holds

(28) $$\frac{1}{\varepsilon_{3}}\lambda_{n}\beta_{n}-\mu \left(1-\varepsilon_{2}\right)\leq -t.$$

Remark 2.4

- Since $0<\alpha <\frac{1}{3}$, one can always find $\varepsilon_{1},\varepsilon_{2}>0$ such that $\varepsilon_{2}<1-4\varepsilon_{1}-\alpha -(\alpha^{2}/4\varepsilon_{1})$ . One possible choice is $\varepsilon_{1}=\frac{\alpha }{4}$ and $0<\varepsilon_{2}<1-3\alpha$. From the second inequality in $\left(C_{4}\right)$ it follows that $1-3\varepsilon_{1}-\varepsilon_{2}>\varepsilon_{1}+\alpha +(\alpha^{2}/4\varepsilon_{1})>0$.
- As
  $$\begin{array}{rl} & 1-\frac{\varepsilon_{1}}{1-3\varepsilon_{1}-\varepsilon_{2}}{\left(1+\frac{\alpha }{2\varepsilon_{1}}\right)}^{2}\\ & \;=\frac{1}{1-3\varepsilon_{1}-\varepsilon_{2}}\left(1-4\varepsilon_{1}-\varepsilon_{2}-\alpha -\frac{\alpha^{2}}{4\varepsilon_{1}}\right)>0, \end{array}$$
  it is always possible to choose *s* such that $0<s\leq 1-\frac{\varepsilon_{1}}{1-3\varepsilon_{1}-\varepsilon }(1+\frac{\alpha }{2\varepsilon_{1}}{)}^{2}$. Since in this case $s<1-4\varepsilon_{1}-\varepsilon_{2}-\alpha -\frac{\alpha^{2}}{4\varepsilon_{1}}$, one has (27).

The following proposition brings us closer to the convergence result.

Proposition 2.5 Let $0<\alpha <\frac{1}{3}$, $\varepsilon_{1},\varepsilon_{2},\varepsilon_{3}>0$ and the sequences $(\lambda_{n}{)}_{n\geq 1}$ and $(\beta_{n}{)}_{n\geq 1}$ satisfy condition $\left(C_{4}\right)$. Let $(x_{n}{)}_{n\geq 0}$ be the sequence generated by Algorithm 2.2 and assume that the Hypotheses 2.2 are verified. Then the following statements are true:

- the sequence $(∥x_{n+1}-x_{n}∥{)}_{n\geq 0}$ belongs to $\ell^{2}$ and the sequence $(\lambda_{n}\beta_{n}∥Bx_{n}{∥}^{2}{)}_{n\geq 1}$ belongs to $\ell^{1}$;
- if, moreover, $\underset{n\to +\infty }{lim inf}\lambda_{n}\beta_{n}>0$, then $\lim_{n\to +\infty }∥Bx_{n}∥=0$ and thus every cluster point of the sequence $(x_{n}{)}_{n\geq 0}$ lies in *M*.
- for every $u\in \mathrm{Zer}(A+D+N_{M})$, the limit $\lim_{n\to +\infty }∥x_{n}-u∥$ exists.

Proof. Since $\lim_{n\to +\infty }\lambda_{n}=0$, there exists a integer $n_{1}\geq 1$ such that $\lambda_{n}\leq (2/\varepsilon_{2})\eta$ for all $n\geq n_{0}$. According to Lemma 2.3, for every $\left(u,y)\in \mathrm{Gr}(A+D+N_{M}\right)$ such that *y*=*v*+*Du*+*p*, with v∈Au and $p\in N_{M}(u)$, and all $n\geq n_{0}$ the following inequality holds

(29) $$\begin{array}{rl} & {∥x_{n+1}-u∥}^{2}-{∥x_{n}-u∥}^{2}\\ & \leq \;\alpha_{n}{∥x_{n}-u∥}^{2}-\alpha_{n}{∥x_{n-1}-u∥}^{2}-\left(1-4\varepsilon_{1}-\varepsilon_{2}\right){∥x_{n+1}-x_{n}∥}^{2}\\ & \;+\left(\alpha_{n}+\frac{\alpha_{n}^{2}}{4\varepsilon_{1}}\right){∥x_{n}-x_{n-1}∥}^{2}+\left(\frac{2}{\varepsilon_{2}}\lambda_{n}\beta_{n}-2\mu \left(1-\varepsilon_{3}\right)\right)\lambda_{n}\beta_{n}{∥Bx_{n}∥}^{2}\\ & \;+\frac{4}{\varepsilon_{2}}\lambda_{n}^{2}{∥Du+v∥}^{2}+2\varepsilon_{3}\lambda_{n}\beta_{n}\left[\underset{u\in M}{\sup}\phi_{B}\left(u,\frac{p}{\varepsilon_{3}\beta_{n}}\right)-\sigma_{M}\left(\frac{p}{\varepsilon_{3}\beta_{n}}\right)\right]\\ & \;+2\lambda_{n}\left\langle u-x_{n},y\right\rangle . \end{array}$$

We consider $u\in \mathrm{Zer}(A+D+N_{M})$, which means that we can take *y*=0 in (29). For all n≥1 we denote

(30) $$\theta_{n}:={∥x_{n}-u∥}^{2},\;\rho_{n}:=\theta_{n}-\alpha_{n}\theta_{n-1}+\left(\alpha_{n}+\frac{\alpha_{n}^{2}}{4\varepsilon_{1}}\right){∥x_{n}-x_{n-1}∥}^{2}$$

and

(31) $$\delta_{n}:=\frac{4}{\varepsilon_{2}}\lambda_{n}^{2}{∥Du+v∥}^{2}+2\varepsilon_{3}\lambda_{n}\beta_{n}\left[\underset{u\in M}{\sup}\phi_{B}\left(u,\frac{p}{\varepsilon_{3}\beta_{n}}\right)-\sigma_{M}\left(\frac{p}{\varepsilon_{3}\beta_{n}}\right)\right].$$

Using that $(\alpha_{n}{)}_{n\geq 1}$ is non-decreasing, for all $n\geq n_{0}$ it yields

(32) $$\begin{array}{rl} \rho_{n+1}-\rho_{n} & \leq \left(\alpha_{n+1}+\frac{\alpha_{n+1}^{2}}{4\varepsilon_{1}}-\left(1-4\varepsilon_{1}-\varepsilon_{2}\right)\right){∥x_{n+1}-x_{n}∥}^{2}\\ & \;+\left(\frac{2}{\varepsilon_{3}}\lambda_{n}\beta_{n}-2\mu \left(1-\varepsilon_{2}\right)\right)\lambda_{n}\beta_{n}{∥Bx_{n}∥}^{2}+\delta_{n}\\ & \leq -s{∥x_{n+1}-x_{n}∥}^{2}-2t\lambda_{n}\beta_{n}{∥Bx_{n}∥}^{2}+\delta_{n}, \end{array}$$

where *s*,*t*>0 are chosen according to (27) and (28), respectively.

Thanks to ($H_{2}^{\mathrm{fitz}}$) and (C${}_{1}$) it holds

(33) $$\begin{array}{rl} \sum_{n\geq 1}\delta_{n} & =\frac{4}{\varepsilon_{2}}{∥Du+v∥}^{2}\sum_{n\geq 1}\lambda_{n}^{2}+2\sum_{n\geq 1}\varepsilon_{3}\lambda_{n}\beta_{n}\left[\underset{u\in M}{\sup}\phi_{B}\left(u,\frac{p}{\varepsilon_{3}\beta_{n}}\right)\right.\\ & \;\left.-\sigma_{M}\left(\frac{p}{\varepsilon_{3}\beta_{n}}\right)\right]<+\infty . \end{array}$$

Hence, according to Lemma 1.4, we obtain

(34) $$\sum_{n\geq 0}{∥x_{n+1}-x_{n}∥}^{2}<+\infty \;\mathrm{and}\;\sum_{n\geq 1}\lambda_{n}\beta_{n}{∥Bx_{n}∥}^{2}<+\infty ,$$

which proves (i). If, in addition, $\underset{n\to \infty }{lim inf}\lambda_{n}\beta_{n}>0$, then $\lim_{n\to +\infty }∥Bx_{n}∥=0$, which means every cluster point of the sequence $(x_{n}{)}_{n\geq 0}$ lies in Zer B=M.

In order to prove (iii), we consider again the inequality (29) for an arbitrary element $u\in \mathrm{Zer}(A+D+N_{M})$ and *y*=0. With the notations in (30) and (31), we get for all $n\geq n_{0}$

(35) $$\theta_{n+1}-\theta_{n}\leq \alpha_{n}\left(\theta_{n}-\theta_{n-1}\right)+\left(\alpha_{n}+\frac{\alpha_{n}^{2}}{4\varepsilon_{1}}\right){∥x_{n}-x_{n-1}∥}^{2}+\delta_{n}.$$

According to (33) and (34) we have

(36) $$\begin{array}{rl} & \sum_{n\geq 1}\left(\alpha_{n}+\frac{\alpha_{n}^{2}}{4\varepsilon_{1}}\right){∥x_{n}-x_{n-1}∥}^{2}+\sum_{n\geq 1}\delta_{n}\leq \left(\alpha +\frac{\alpha^{2}}{4\varepsilon_{1}}\right)\sum_{n\geq 1}{∥x_{n}-x_{n-1}∥}^{2}\\ & \;+\sum_{n\geq 1}\delta_{n}<+\infty , \end{array}$$

therefore, by Lemma 1.2, the limit $\lim_{n\to +\infty }\theta_{n}=\lim_{n\to +\infty }∥x_{n}-u{∥}^{2}$ exists, which means that the limit $\lim_{n\to +\infty }∥x_{n}-u∥$ exists, too.

Remark 2.6 The condition (C${}_{3}$) that we imposed in combination with $0<\alpha <\frac{1}{3}$ on the sequence of inertial parameters $(\alpha_{n}{)}_{n\geq 1}$ is the one proposed in [15, Proposition 2.1] when addressing the convergence of the inertial proximal point algorithm. However, the statements in the proposition above and in the following convergence theorem remain valid if one alternatively assumes that there exists $\alpha^{\prime }$ such that $0\leq \alpha_{n}\leq \alpha^{\prime }<1$ for all n≥1 and

$$\sum_{n\geq 1}\left(\alpha_{n}+\frac{\alpha_{n}^{2}}{4\varepsilon_{1}}\right){∥x_{n}-x_{n-1}∥}^{2}<+\infty .$$

This can be realized if one chooses for a fixed *p*>1

$$\alpha_{n}\leq \min\left\{\alpha^{\prime },2\varepsilon_{1}\left(-1+\sqrt{1+n^{-p}{∥x_{n}-x_{n-1}∥}^{-2}}\right)\right\}\;\forall n\geq 1.$$

Indeed, in this situation we have that $(\alpha_{n}^{2}/4\varepsilon_{1})+\alpha_{n}-(1/n^{p}∥x_{n}-x_{n-1}{∥}^{2})\leq \;0$ for all n≥1, which gives

$$\sum_{n\geq 1}\left(\alpha_{n}+\frac{\alpha_{n}^{2}}{4\varepsilon_{1}}\right){∥x_{n}-x_{n-1}∥}^{2}\leq \sum_{n\geq 1}\frac{1}{n^{p}}<+\infty .$$

Now we are ready to prove the main theorem of this section, which addresses the convergence of the sequence generated by Algorithm 2.2.

Theorem 2.8 Let $0<\alpha <\frac{1}{3}$, $\varepsilon_{1},\varepsilon_{2},\varepsilon_{3}>0$ and the sequences $(\lambda_{n}{)}_{n\geq 1}$ and $(\beta_{n}{)}_{n\geq 1}$ satisfy condition $\left(C_{4}\right)$. Let $(x_{n}{)}_{n\geq 0}$ be the sequence generated by Algorithm 2.2, $(z_{n}{)}_{n\geq 1}$ be the sequence defined in (7) and assume that the Hypotheses 2.2 are verified. Then the following statements are true:

- the sequence $(z_{n}{)}_{n\geq 1}$ converges weakly to an element in $\mathrm{Zer}(A+D+N_{M})$ as n→+∞.
- if *A* is *γ*-strongly monotone with γ>0, then $(x_{n}{)}_{n\geq 0}$ converges strongly to the unique element in $\mathrm{Zer}(A+D+N_{M})$ as n→+∞.

Proof.

1. According to Proposition 2.5 (iii), the limit $\lim_{n\to +\infty }∥x_{n}-u∥$ exists for every $u\in \mathrm{Zer}(A+D+N_{M})$. Let *z* be a sequential weak cluster point of $(z_{n}{)}_{n\geq 1}$. We will show that $z\in \mathrm{Zer}(A+D+N_{M})$, by using the characterization (5) of the maximal monotonicity, and the conclusion will follow by Lemma 1.1. To this end we consider an arbitrary $\left(u,y)\in \mathrm{Gr}(A+D+N_{M}\right)$ such that *y*=*v*+*Du*+*p*, where v∈Au and $p\in N_{M}(u)$. From (29), with the notations (30) and (31), we have for all $n\geq n_{0}$
  (37) $$\begin{array}{rl} & \rho_{n+1}-\rho_{n}\\ & \;\leq -s{∥x_{n+1}-x_{n}∥}^{2}-2t\lambda_{n}\beta_{n}{∥Bx_{n}∥}^{2}+\delta_{n}+2\lambda_{n}\left\langle u-x_{n},y\right\rangle \\ & \;\leq \delta_{n}+2\lambda_{n}\left\langle u-x_{n},y\right\rangle . \end{array}$$
  Recall that from (33) that $\sum_{n\geq 1}\delta_{n}<+\infty$. Since $(x_{n}{)}_{n\geq 0}$ is bounded, the sequence $(\rho_{n}{)}_{n\geq 1}$ is also bounded.
  We fix an arbitrary integer $\bar{N}\geq n_{0}$ and sum up the inequalities in (37) for $n=n_{0}+1,n_{0}+2,\ldots ,\bar{N}$. This yields
  $$\begin{array}{rl} \rho_{\bar{N}+1}-\rho_{n_{0}+1} & \leq \sum_{n\geq 1}\delta_{n}+2\left\langle -\sum_{n=1}^{n_{0}}\lambda_{n}u+\sum_{n=1}^{n_{0}}\lambda_{n}x_{n},y\right\rangle \\ & \;+2\left\langle \tau_{\bar{N}}u-\sum_{n=1}^{\bar{N}}\lambda_{n}x_{n},y\right\rangle . \end{array}$$
  After dividing this last inequality by $2\tau_{\bar{N}}=2\sum_{n=1}^{\bar{N}}\lambda_{n}$, we obtain
  (38) $$\frac{1}{2\tau_{\bar{N}}}\left(\rho_{\bar{N}+1}-\rho_{n_{0}+1}\right)\leq \frac{1}{2\tau_{\bar{N}}}T+2\left\langle u-z_{\bar{N}},y\right\rangle ,$$
  where $T:=\sum_{n\geq 1}\delta_{n}+2\langle -\sum_{n=1}^{n_{0}}\lambda_{n}u+\sum_{n=1}^{n_{0}}\lambda_{n}x_{n},y\rangle \in R$. By passing in (38) to the limit and by using that $\lim_{N\to \infty }\tau_{\bar{N}}=\lim_{\bar{N}\to \infty }\sum_{n=1}^{\bar{N}}\lambda_{n}=+\infty$, we get
  $$\underset{\bar{N}\to \infty }{lim inf}\left\langle u-z_{\bar{N}},y\right\rangle \geq 0.$$
  As *z* is a sequential weak cluster point of $(z_{n}{)}_{n\geq 1}$, the above inequality gives us ⟨u−z,y⟩≥0, which finally means that $z\in \mathrm{Zer}(A+D+N_{M})$.
2. Let u∈H be the unique element in $\mathrm{Zer}(A+D+N_{M})$. Since *A* is γ−strongly monotone with γ>0, the formula in (15) reads for all n≥1
  $$\begin{array}{rl} & \left\langle x_{n+1}-u,x_{n}-x_{n+1}-\lambda_{n}\left(Dx_{n}+\beta_{n}Bx_{n}+v\right)+\alpha_{n}\left(x_{n}-x_{n-1}\right)\right\rangle \\ & \;\geq \gamma \lambda_{n}{∥x_{n+1}-u∥}^{2} \end{array}$$
  or, equivalently,
  $$\begin{array}{rl} & 2\gamma \lambda_{n}{∥x_{n+1}-u∥}^{2}+2\left\langle u-x_{n+1},x_{n}-x_{n+1}\right\rangle \\ & \;\leq \;2\lambda_{n}\left\langle u-x_{n+1},\beta_{n}Bx_{n}+Dx_{n}+v\right\rangle -2\alpha_{n}\left\langle u-x_{n+1},x_{n}-x_{n-1}\right\rangle . \end{array}$$
  By using again (17), (18) and (26) we obtain for all n≥1
  $$\begin{array}{rl} & 2\gamma \lambda_{n}{∥x_{n+1}-u∥}^{2}+{∥x_{n+1}-u∥}^{2}-{∥x_{n}-u∥}^{2}\\ & \;\leq \alpha_{n}{∥x_{n}-u∥}^{2}-\alpha_{n}{∥x_{n-1}-u∥}^{2}-\left(1-4\varepsilon_{1}-\varepsilon_{2}\right){∥x_{n+1}-x_{n}∥}^{2}\\ & \;+\left(\alpha_{n}+\frac{\alpha_{n}^{2}}{4\varepsilon_{1}}\right){∥x_{n}-x_{n-1}∥}^{2}\;+\;\left(\frac{2}{\varepsilon_{2}}\lambda_{n}^{2}\beta_{n}^{2}-2\mu \left(1\;-\;\varepsilon_{3}\right)\lambda_{n}\beta_{n}\right){∥Bx_{n}∥}^{2}\\ & \;+\left(\frac{4}{\varepsilon_{2}}\lambda_{n}^{2}-2\eta \lambda_{n}\right){∥Dx_{n}-Du∥}^{2}+\frac{4}{\varepsilon_{2}}\lambda_{n}^{2}{∥Du+v∥}^{2}\\ & \;+2\varepsilon_{3}\lambda_{n}\beta_{n}\left[\underset{u\in M}{\sup}\phi_{B}\left(u,\frac{p}{\varepsilon_{3}\beta_{n}}\right)-\sigma_{M}\left(\frac{p}{\varepsilon_{3}\beta_{n}}\right)\right]+2\lambda_{n}\left\langle u-x_{n},y\right\rangle . \end{array}$$
  By using the notations in (30) and (31), this yields for all n≥1
  $$\begin{array}{rl} & 2\gamma \lambda_{n}{∥x_{n+1}-u∥}^{2}+\theta_{n+1}-\theta_{n}\leq \alpha_{n}\left(\theta_{n}-\theta_{n-1}\right)\\ & \;+\left(\alpha_{n}+\frac{\alpha_{n}^{2}}{4\varepsilon_{1}}\right){∥x_{n}-x_{n-1}∥}^{2}+\delta_{n}. \end{array}$$
  By taking into account (36), from Lemma 1.2 we get
  $$2\gamma \sum_{n\geq 1}\lambda_{n}{∥x_{n}-u∥}^{2}<+\infty .$$
  According to (C${}_{1}$) we have $\sum_{n\geq 1}\lambda_{n}=+\infty$, which implies that the limit $\lim_{n\to \infty }∥x_{n}-u∥$ must be equal to zero. This provides the desired conclusion.

## 3. Applications to convex bilevel programming

We will employ the results obtained in the previous section, in the context of monotone inclusions, to the solving of convex bilevel programming problems.

Problem 3.1 Let H be a real Hilbert space, $f:H\to \bar{R}$ a proper, convex and lower semicontinuous function and g,h:H→R differentiable functions with $L_{g}$-Lipschitz continuous and, respectively, $L_{h}$-Lipschitz continuous gradients. Suppose that arg⁡minh≠∅ and minh=0. The bilevel programming problem to solve reads

$$\min_{x\in \arg\min h}f\left(x\right)+g\left(x\right).$$

The assumption minh=0 is not restrictive as, otherwise, one can replace *h* with h−minh.

Hypotheses 3.2 The convergence analysis will be carry out in the following hypotheses:

1. $\partial f+N_{\arg\min h}\;\mathrm{is}\;\mathrm{maximally}\;\mathrm{monotone}\;\mathrm{and}\;S:=\arg\min_{x\in \arg\min h}\{f(x)+g(x)\}\neq \emptyset$;
2. for every $p\in \mathrm{Ran}N_{\arg\min h},\sum_{n\geq 1}\lambda_{n}\beta_{n}[h^{*}(\frac{p}{\beta_{n}})-\sigma_{\arg\min h}(\frac{p}{\beta_{n}})]<+\infty$.

In the above hypotheses, we have that $\partial f+\nabla g+N_{\arg\min h}=\partial (f+g+\delta_{\arg\min h})$ and hence $S=\mathrm{Zer}(\partial f+\nabla g+N_{\arg\min h})\neq \emptyset$. Since according to the Theorem of Baillon–Haddad (see, e.g. [33, Corollary 18.16]), ∇g and ∇h are $L_{g}^{-1}$-cocoercive and, respectively, $L_{h}^{-1}$- cocoercive, and arg⁡minh=Zer∇h, solving the bilevel programming problem in Problem 3.1 reduces to solving the monotone inclusion

$$0\in \partial f(x)+\nabla g(x)+N_{\arg\min h}(x).$$

By using to this end Algorithm 2.2, we receive the following iterative scheme.


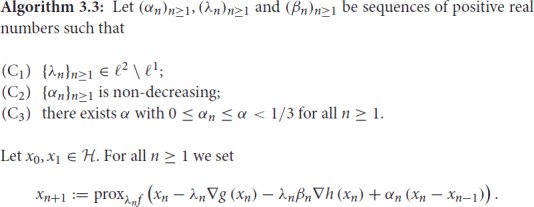


By using the inequality (6), one can easily notice, that $\left(H_{2}^{\mathrm{prog}}\right)$ implies $\left(H_{2}^{\mathrm{fitz}}\right)$, which means that the convergence statements for Algorithm 3.3 can be derived as particular instances of the ones derived in the previous section.

Alternatively, one can use to this end the following lemma and employ the same ideas and techniques as in Section 2. Lemma 3.3 is similar to Lemma 2.3, however, it will allow us to provide convergence statements also for the sequence of function values $(h(x_{n}){)}_{n\geq 0}$.

Lemma 3.3 Let $(x_{n}{)}_{n\geq 0}$ be the sequence generated by *Algorithm 3.3* and (u,y) be an element in $\mathrm{Gr}(\partial f+\nabla g+N_{\arg\min h})$ such that y=v+∇g(u)+p with v∈∂f(u) and $p\in N_{\arg\min h}(u)$. Further, let $\varepsilon_{1},\varepsilon_{2},\varepsilon_{3}>0$ be such that $1-\varepsilon_{3}>0$. Then the following inequality holds for all n≥1

$$\begin{array}{rl} & {∥x_{n+1}-u∥}^{2}-{∥x_{n}-u∥}^{2}\\ & \;\leq \;\alpha_{n}{∥x_{n}-u∥}^{2}-\alpha_{n}{∥x_{n-1}-u∥}^{2}-\left(1-4\varepsilon_{1}-\varepsilon_{2}\right){∥x_{n+1}-x_{n}∥}^{2}\\ & \;+\left(\alpha_{n}+\frac{\alpha_{n}^{2}}{4\varepsilon_{1}}\right){∥x_{n}-x_{n-1}∥}^{2}\left(\frac{2}{\varepsilon_{2}}\lambda_{n}^{2}\beta_{n}^{2}-2\mu \left(1-\varepsilon_{3}\right)\lambda_{n}\beta_{n}\right){∥\nabla h\left(x_{n}\right)∥}^{2}\\ & \;+\left(\frac{4}{\varepsilon_{2}}\lambda_{n}^{2}-2\eta \lambda_{n}\right){∥\nabla g\left(x_{n}\right)-\nabla g\left(u\right)∥}^{2}+\lambda_{n}\beta_{n}\left[h\left(u\right)-h\left(x_{n}\right)\right]\\ & \;+\frac{4}{\varepsilon_{2}}\lambda_{n}^{2}{∥v+\nabla g\left(u\right)∥}^{2}+\varepsilon_{3}\lambda_{n}\beta_{n}\left[h^{*}\left(\frac{2p}{\varepsilon_{3}\beta_{n}}\right)-\sigma_{\arg\min h}\left(\frac{2p}{\varepsilon_{3}\beta_{n}}\right)\right]\\ & \;+2\lambda_{n}\left\langle u-x_{n},y\right\rangle . \end{array}$$

Proof. Let be n≥1 fixed. The proof follows by combining the estimates used in the proof of Lemma 2.3 with some inequalities which better exploit the convexity of *h*. From (23) we have

$$\begin{array}{rl} 2\lambda_{n}\beta_{n}\left\langle u-x_{n},\nabla h\left(x_{n}\right)\right\rangle & \leq -2\mu \left(1-\varepsilon_{3}\right)\lambda_{n}\beta_{n}{∥\nabla h\left(x_{n}\right)∥}^{2}\\ & \;+2\varepsilon_{3}\lambda_{n}\beta_{n}\left\langle u-x_{n},\nabla h\left(x_{n}\right)\right\rangle . \end{array}$$

Since *h* is convex, the following relation also holds

$$2\lambda_{n}\beta_{n}\left\langle u-x_{n},\nabla h\left(x_{n}\right)\right\rangle \leq 2\lambda_{n}\beta_{n}\left[h\left(u\right)-h\left(x_{n}\right)\right].$$

Summing up the two inequalities above gives

$$\begin{array}{rl} & 2\lambda_{n}\beta_{n}\left\langle u-x_{n},\nabla h\left(x_{n}\right)\right\rangle \leq -\mu \left(1-\varepsilon_{3}\right)\lambda_{n}\beta_{n}{∥\nabla h\left(x_{n}\right)∥}^{2}\\ & \;+\varepsilon_{3}\lambda_{n}\beta_{n}\left\langle u-x_{n},\nabla h\left(x_{n}\right)\right\rangle +\lambda_{n}\beta_{n}\left[h\left(u\right)-h\left(x_{n}\right)\right]. \end{array}$$

Using the same techniques as in the derivation of (25), we get

$$\begin{array}{rl} & 2\lambda_{n}\left\langle u-x_{n},v+\nabla g\left(x_{n}\right)+\beta_{n}\nabla h\left(x_{n}\right)\right\rangle \\ & \;\leq -\mu \left(1-\varepsilon_{3}\right)\lambda_{n}\beta_{n}{∥\nabla h\left(x_{n}\right)∥}^{2}-2\eta \lambda_{n}{∥\nabla g\left(x_{n}\right)-\nabla g\left(u\right)∥}^{2}\\ & \;+\lambda_{n}\beta_{n}\left[h\left(u\right)-h\left(x_{n}\right)\right]+2\lambda_{n}\left\langle u-x_{n},y\right\rangle \\ & \;+\varepsilon_{3}\lambda_{n}\beta_{n}\left[h^{*}\left(u,\frac{2p}{\varepsilon_{3}\beta_{n}}\right)-\sigma_{\arg\min h}\left(\frac{2p}{\varepsilon_{3}\beta_{n}}\right)\right]. \end{array}$$

With these improved estimates, the conclusion follows as in the proof of Lemma 2.3.

By using now Lemma 3.3, one obtains, after slightly adapting the proof of Proposition 2.5, the following result.

Proposition 3.4 Let $0<\alpha <\frac{1}{3}$, $\varepsilon_{1},\varepsilon_{2},\varepsilon_{3}>0$ and the sequences $(\lambda_{n}{)}_{n\geq 1}$ and $(\beta_{n}{)}_{n\geq 1}$ satisfy condition $\left(C_{4}\right)$. Let $(x_{n}{)}_{n\geq 0}$ be the sequence generated by Algorithm 3.3 and assume that the Hypotheses 3.2 are verified. Then the following statements are true:

- the sequence $(∥x_{n+1}-x_{n}∥{)}_{n\geq 0}$ belongs to $\ell^{2}$ and the sequences $(\lambda_{n}\beta_{n}∥\nabla h(x_{n}){∥}^{2}{)}_{n\geq 1}$ and $(\lambda_{n}\beta_{n}h(x_{n}){)}_{n\geq 1}$ belong to $\ell^{1}$;
- if, moreover, $\underset{n\to +\infty }{lim inf}\lambda_{n}\beta_{n}>0$, then $\lim_{n\to +\infty }∥\nabla h(x_{n})∥\;=\;\lim_{n\to +\infty }h(x_{n})=0$ and thus every cluster point of the sequence $(x_{n}{)}_{n\geq 0}$ lies in arg⁡minh.
- for every u∈S, the limit $\lim_{n\to +\infty }∥x_{n}-u∥$ exists.

Finally, the above proposition leads to the following convergence result.

Theorem 3.6 Let $0<\alpha <\frac{1}{3}$, $\varepsilon_{1},\varepsilon_{2},\varepsilon_{3}>0$ and the sequences $(\lambda_{n}{)}_{n\geq 1}$ and $(\beta_{n}{)}_{n\geq 1}$ satisfy condition $\left(C_{4}\right)$. Let $(x_{n}{)}_{n\geq 0}$ be the sequence generated by Algorithm 3.3, $(z_{n}{)}_{n\geq 1}$ be the sequence defined in (7) and assume that the Hypotheses 3.2 are verified. Then the following statements are true:

- the sequence $(z_{n}{)}_{n\geq 1}$ converges weakly to an element in S as n→+∞.
- if *f* is γ−strongly convex with γ>0, then $(x_{n}{)}_{n\geq 0}$ converges strongly to the unique element in S as n→+∞.

As follows we will show that under inf-compactness assumptions one can achieve weak non-ergodic convergence for the sequence $(x_{n}{)}_{n\geq 0}$. Weak non-ergodic convergence has been obtained for Algorithm 3.3 in [9] when $\alpha_{n}=\alpha$ for all n≥1 and for restrictive choices for both the sequence of step sizes and penalty parameters.

We denote by $\left(f+g{)}_{*}=\min_{x\in \arg\min h}(f(x)+g(x)\right)$. For every element *x* in H, we denote by $\mathrm{dist}(x,S)=\underset{u\in S}{\inf}∥x-u∥$ the distance from *x* to S. In particular, $\mathrm{dist}(x,S)=∥x-{Pr}_{S}x∥$, where ${Pr}_{S}x$ denotes the projection of *x* onto S. The projection operator ${Pr}_{S}$ is *firmly non-expansive* [33, Proposition 4.8], this means

(39) $$\begin{array}{rl} & {∥{Pr}_{S}\left(x\right)-{Pr}_{S}\left(y\right)∥}^{2}+{∥\left[\mathrm{Id}-{Pr}_{S}\right]\left(x\right)-\left[\mathrm{Id}-{Pr}_{S}\right]\left(y\right)∥}^{2}\\ & \;\leq {∥x-y∥}^{2}\;\forall x,y\in H. \end{array}$$

Denoting $d(x)=\frac{1}{2}\mathrm{dist}(x,S{)}^{2}=\frac{1}{2}∥x-{Pr}_{S}x{∥}^{2}$ for all x∈H, one has that x↦d(x) is differentiable and it holds $\nabla d(x)=x-{Pr}_{S}x$ for all x∈H.

Lemma 3.6 Let $(x_{n}{)}_{n\geq 0}$ be the sequence generated by Algorithm 3.3 and assume that the Hypotheses 3.2 are verified. Then the following inequality holds for all n≥1

(40) $$\begin{array}{rl} & d\left(x_{n+1}\right)-d\left(x_{n}\right)-\alpha_{n}\left(d\left(x_{n}\right)-d\left(x_{n-1}\right)\right)+\lambda_{n}\left[(f+g)\left(x_{n+1}\right)-(f+g{)}_{*}\right]\\ & \;\leq \;\left(\frac{L_{g}}{2}\lambda_{n}+\frac{L_{h}}{4}\lambda_{n}\beta_{n}+\frac{\alpha_{n}}{2}\right){∥x_{n+1}-x_{n}∥}^{2}+\alpha_{n}{∥x_{n}-x_{n-1}∥}^{2}. \end{array}$$

Proof. Let n≥1 be fixed. Since *d* is convex, we have

(41) $$d\left(x_{n+1}\right)-d\left(x_{n}\right)\leq \left\langle x_{n+1}-{Pr}_{S}\left(x_{n+1}\right),x_{n+1}-x_{n}\right\rangle .$$

Then there exists $v_{n+1}\in \partial f(x_{n+1})$ such that (see (14))

$$x_{n}-x_{n+1}-\lambda_{n}(\nabla g(x_{n})+\beta_{n}\nabla h(x_{n}))+\alpha_{n}(x_{n}-x_{n-1})=\lambda_{n}v_{n+1}$$

and, so,

(42) $$\begin{array}{rl} & \left\langle x_{n+1}-{Pr}_{S}\left(x_{n+1}\right),x_{n+1}-x_{n}\right\rangle \\ & \;=\;\left\langle x_{n+1}-{Pr}_{S}\left(x_{n+1}\right),-\lambda_{n}v_{n+1}-\lambda_{n}\nabla g\left(x_{n}\right)-\lambda_{n}\beta_{n}\nabla h\left(x_{n}\right)+\alpha_{n}\left(x_{n}\;-\;x_{n-1}\right)\right\rangle \\ & \;-\lambda_{n}\beta_{n}\left\langle x_{n+1}-{Pr}_{S}\left(x_{n+1}\right),\nabla h\left(x_{n}\right)\right\rangle +\alpha_{n}\left\langle x_{n+1}-{Pr}_{S}\left(x_{n+1}\right),x_{n}-x_{n-1}\right\rangle . \end{array}$$

Since $v_{n+1}\in \partial f(x_{n+1})$, we get

(43) $$-\lambda_{n}\left\langle x_{n+1}-{Pr}_{S}\left(x_{n+1}\right),v_{n+1}\right\rangle \leq \lambda_{n}\left[f\left({Pr}_{S}\left(x_{n+1}\right)\right)-f\left(x_{n+1}\right)\right].$$

Using the convexity of *g* it follows

(44) $$g\left(x_{n}\right)-g\left({Pr}_{S}\left(x_{n+1}\right)\right)\leq \left\langle \nabla g\left(x_{n}\right),x_{n}-{Pr}_{S}\left(x_{n+1}\right)\right\rangle .$$

On the other hand, the Descent Lemma gives

(45) $$g\left(x_{n+1}\right)\leq g\left(x_{n}\right)+\left\langle \nabla g\left(x_{n}\right),x_{n+1}-x_{n}\right\rangle +\frac{L_{g}}{2}{∥x_{n+1}-x_{n}∥}^{2}.$$

By adding (44) and (45), it yields

(46) $$\begin{array}{rl} & -\lambda_{n}\left\langle x_{n+1}-{Pr}_{S}\left(x_{n+1}\right),\nabla g\left(x_{n}\right)\right\rangle \leq \;\lambda_{n}\left[g\left({Pr}_{S}\left(x_{n+1}\right)\right)-g\left(x_{n+1}\right)\right]\\ & \;+\frac{L_{g}\lambda_{n}}{2}{∥x_{n+1}-x_{n}∥}^{2}. \end{array}$$

Using the $(1/L_{h})-$ cocoercivity of ∇h combined with the fact that $\nabla h({Pr}_{S}(x_{n+1}))=0$ (as ${Pr}_{S}(x_{n+1})$ belongs to S), it yields

$$-\left\langle x_{n}-{Pr}_{S}\left(x_{n+1}\right),\nabla h\left(x_{n}\right)\right\rangle \leq -\frac{1}{L_{h}}{∥\nabla h\left(x_{n}\right)∥}^{2}.$$

Therefore

(47) $$\begin{array}{rl} & -\lambda_{n}\beta_{n}\left\langle x_{n+1}-{Pr}_{S}\left(x_{n+1}\right),\nabla h\left(x_{n}\right)\right\rangle \\ & \;\leq \lambda_{n}\beta_{n}\left(\left\langle x_{n}-x_{n+1},\nabla h\left(x_{n}\right)\right\rangle -\frac{1}{L_{h}}{∥\nabla h\left(x_{n}\right)∥}^{2}\right)\\ & \;\leq \lambda_{n}\beta_{n}\frac{L_{h}}{4}{∥x_{n+1}-x_{n}∥}^{2}. \end{array}$$

Further, we have

$$\begin{array}{rl} & \alpha_{n}\left\langle x_{n+1}-{Pr}_{S}\left(x_{n+1}\right)-\left(x_{n}-{Pr}_{S}\left(x_{n}\right)\right),x_{n}-x_{n-1}\right\rangle \\ & \;\leq \;\frac{\alpha_{n}}{2}{∥\left[\mathrm{Id}-{Pr}_{S}\right]\left(x_{n+1}\right)-\left[\mathrm{Id}-{Pr}_{S}\right]\left(x_{n}\right)∥}^{2}+\frac{\alpha_{n}}{2}{∥x_{n}-x_{n-1}∥}^{2}\\ & \;\leq \frac{\alpha_{n}}{2}{∥x_{n+1}-x_{n}∥}^{2}+\frac{\alpha_{n}}{2}{∥x_{n}-x_{n-1}∥}^{2}, \end{array}$$

and

$$\begin{array}{rl} & \alpha_{n}\left\langle x_{n}-{Pr}_{S}\left(x_{n}\right),x_{n}-x_{n-1}\right\rangle \\ & \;=\alpha_{n}d\left(x_{n}\right)+\frac{\alpha_{n}}{2}{∥x_{n}-x_{n-1}∥}^{2}-\frac{\alpha_{n}}{2}{∥x_{n-1}-{Pr}_{S}\left(x_{n}\right)∥}^{2}\\ & \;\leq \alpha_{n}d\left(x_{n}\right)+\frac{\alpha_{n}}{2}{∥x_{n}-x_{n-1}∥}^{2}-\alpha_{n}d\left(x_{n-1}\right). \end{array}$$

By adding two relations above, we obtain

(48) $$\begin{array}{rl} & \alpha_{n}\left\langle x_{n+1}-{Pr}_{S}\left(x_{n+1}\right),x_{n}-x_{n-1}\right\rangle \\ & \;=\;\alpha_{n}\left\langle x_{n+1}-{Pr}_{S}\left(x_{n+1}\right)-\left(x_{n}-{Pr}_{S}\left(x_{n}\right)\right),x_{n}-x_{n-1}\right\rangle \\ & \;+\alpha_{n}\left\langle x_{n}-{Pr}_{S}\left(x_{n}\right),x_{n}-x_{n-1}\right\rangle \\ & \;\leq \frac{\alpha_{n}}{2}{∥x_{n+1}-x_{n}∥}^{2}+\alpha_{n}{∥x_{n}-x_{n-1}∥}^{2}+\alpha_{n}\left(d\left(x_{n}\right)-d\left(x_{n-1}\right)\right). \end{array}$$

By combining (43), (46), (47) and (48) with (42) we obtain the desired conclusion.

Definition 3.7 A function $\Psi :H\to \bar{R}$ is sad to be inf-compact if for every *r*>0 and κ∈R the set

$${\mathrm{Lev}}_{\kappa }^{r}\left(\Psi \right):=\left\{x\in H:∥x∥\leq r,\Psi \left(x\right)\leq \kappa \right\}$$

is relatively compact in H.

An useful property of inf-compact functions follows.

Lemma 3.8 Let $\Psi :H\to \bar{R}$ be inf-compact and $(x_{n}{)}_{n\geq 0}$ be a bounded sequence in H such that $(\Psi (x_{n}){)}_{n\geq 0}$ is bounded as well. If the sequence $(x_{n}{)}_{n\geq 0}$ converges weakly to an element in $\hat{x}$ as n→+∞, then it converges strongly to this element.

Proof. Let be $\bar{r}>0$ and $\bar{\kappa }\in R$ such that for all n≥1

$$∥x_{n}∥\leq \bar{r}\;\mathrm{and}\;\Psi \left(x_{n}\right)\leq \bar{\kappa }.$$

Hence, $(x_{n}{)}_{n\geq 0}$ belongs to the set ${\mathrm{Lev}}_{\bar{\kappa }}^{\bar{r}}(\Psi )$, which is relatively compact. Then $(x_{n}{)}_{n\geq 0}$ has at least one strongly convergent subsequence. Since every strongly convergent subsequence $(x_{n_{l}}{)}_{l\geq 0}$ of $(x_{n}{)}_{n\geq 0}$ has as limit $\hat{x}$, the desired conclusion follows.

We can formulate now the weak non-ergodic convergence result.

Theorem 3.10 Let $0<\alpha <\frac{1}{3}$, $\varepsilon_{1},\varepsilon_{2},\varepsilon_{3}>0$, the sequences $(\lambda_{n}{)}_{n\geq 1}$ and $(\beta_{n}{)}_{n\geq 1}$ satisfy the condition $0<\underset{n\to \infty }{lim inf}\lambda_{n}\beta_{n}\leq \underset{n\geq 0}{\sup}\lambda_{n}\beta_{n}\leq \mu$, $(x_{n}{)}_{n\geq 0}$ be the sequence generated by Algorithm 3.3, and assume that the Hypotheses 3.2 are verified and that either *f*+*g* or *h* is inf-compact. Then the following statements are true:

1. $\lim_{n\to +\infty }d(x_{n})=0$;
2. the sequence $(x_{n}{)}_{n\geq 0}$ converges weakly to an element in S as n→+∞;
3. if *h* is inf-compact, then the sequence $(x_{n}{)}_{n\geq 0}$ converges strongly to an element in S as n→+∞.

Proof.

1. Thanks to Lemma 3.6, for all n≥1 we have
  (49) $$\begin{array}{rl} & d\left(x_{n+1}\right)-d\left(x_{n}\right)+\lambda_{n}\left[(f+g)\left(x_{n+1}\right)-(f+g{)}_{*}\right]\\ & \;\leq \alpha_{n}\left(d\left(x_{n}\right)-d\left(x_{n-1}\right)\right)+\zeta_{n}, \end{array}$$
  where
  $$\zeta_{n}:=\;\left(\frac{L_{g}}{2}\lambda_{n}+\frac{L_{h}}{4}\lambda_{n}\beta_{n}+\frac{\alpha_{n}}{2}\right){∥x_{n+1}-x_{n}∥}^{2}+\alpha_{n}{∥x_{n}-x_{n-1}∥}^{2}.$$
  From Proposition 3.4 (i), combined with the fact that both sequences $(\lambda_{n}{)}_{n\geq 1}$ and $(\beta_{n}{)}_{n\geq 1}$ are bounded, it follows that $\sum_{n\geq 1}\zeta_{n}<+\infty$.
  In general, since $(x_{n}{)}_{n\geq 0}$ is not necessarily included in arg⁡minh, we have to treat two different cases.
  *Case 1*: There exists an integer $n_{1}\geq 1$ such that $(f+g)(x_{n})\geq (f+g{)}_{*}$ for all $n\geq n_{1}$. In this case, we obtain from Lemma 1.2 that:
  - the limit $\lim_{n\to +\infty }d(x_{n})$ exists.
  - $\sum_{n\geq n_{2}}\lambda_{n}[(f+g)(x_{n+1})-(f+g{)}_{*}]<+\infty$. Moreover, since $(\lambda_{n}{)}_{n\geq 1}\notin \ell^{1}$, we must have
    (50) $$\underset{n\to +\infty }{lim inf}(f+g)\left(x_{n}\right)\leq (f+g{)}_{*}.$$
  Consider a subsequence $(x_{n_{k}}{)}_{k\geq 0}$ of $(x_{n}{)}_{n\geq 0}$ such that
  $$\lim_{k\to +\infty }(f+g)\left(x_{n_{k}}\right)=\underset{n\to +\infty }{lim inf}(f+g)\left(x_{n}\right)$$
  and note that, thanks to (50), the sequence $((f+g)(x_{n_{k}}){)}_{k\geq 0}$ is bounded. From Proposition 3.4 (ii) –(iii) we get that also $(x_{n_{k}}{)}_{k\geq 0}$ and $(h(x_{n_{k}}){)}_{k\geq 0}$ are bounded. Thus, since either *f*+*g* or *h* is inf-compact, there exists a subsequence $(x_{n_{l}}{)}_{l\geq 0}$ of $(x_{n_{k}}{)}_{k\geq 0}$, which converges strongly to an element $\hat{x}$ as l→+∞. According to Proposition 3.4 (ii) –(iii), $\hat{x}$ belongs to arg⁡minh. On the other hand,
  (51) $$\lim_{l\to +\infty }(f+g)\left(x_{n_{l}}\right)=\underset{n\to +\infty }{lim inf}(f+g)\left(x_{n}\right)\geq (f+g)\left(\hat{x}\right)\geq (f+g{)}_{*}.$$
  We deduce from (50)–(51) that $(f+g)(\hat{x})=(f+g{)}_{*}$, or in other words, that $\hat{x}\in S$. In conclusion, thanks to the continuity of *d*,
  $$\lim_{n\to +\infty }d\left(x_{n}\right)=\lim_{l\to \infty }d\left(x_{n_{l}}\right)=d\left(\hat{x}\right)=0.$$
  *Case 2*: For all n≥1 there exists some $n^{\prime }>n$ such that $(f+g)(x_{n^{\prime }})<(f+g{)}_{*}$. We define the set
  $$V=\left\{n^{\prime }\geq 1:(f+g)\left(x_{n^{\prime }}\right)<(f+g{)}_{*}\right\}.$$
  There exists an integer $n_{2}\geq 2$ such that for all $n\geq n_{2}$ the set {k≤n:k∈V} is non-empty. Hence, for all $n\geq n_{2}$ the number
  $$t_{n}:=\max\left\{k\leq n:k\in V\right\}$$
  is well-defined. By definition $t_{n}\leq n$ for all $n\geq n_{3}$ and moreover the sequence $\{t_{n}{\}}_{n\geq n_{2}}$ is non-decreasing and $\lim_{n\to +\infty }t_{n}=\infty$. Indeed, if $\lim_{n\to \infty }t_{n}=t\in R$, then for all $n^{\prime }>t$ it holds $(f+g)(x_{n^{\prime }})\geq (f+g{)}_{*}$, contradiction. Choose an integer $N\geq n_{2}$.
  - If $t_{N}<N$, then, for all $n=t_{N},\ldots ,N-1$, since $(f+g)(x_{n})\geq (f+g{)}_{*}$, the inequality (49) gives
    (52) $$\begin{array}{rl} & d\left(x_{n+1}\right)-d\left(x_{n}\right)\leq d\left(x_{n+1}\right)-d\left(x_{n}\right)+\lambda_{n}\left[F\left(x_{n+1}\right)-F_{*}\right]\\ & \;\leq \alpha_{n}\left(d\left(x_{n}\right)-d\left(x_{n-1}\right)\right)+\zeta_{n}. \end{array}$$
    Summing (52) for $n=t_{N},\ldots ,N-1$ and using tht $\{\alpha_{n}{\}}_{n\geq 1}$ is non-decreasing, it yields
    (53) $$\begin{array}{rl} & d\left(x_{N}\right)-d\left(x_{t_{N}}\right)\leq \;\sum_{n=t_{N}}^{N-1}\left(\alpha_{n}d\left(x_{n}\right)-\alpha_{n-1}d\left(x_{n-1}\right)\right)+\sum_{n=t_{N}}^{N-1}\zeta_{n}\\ & \;\leq \alpha d\left(x_{N-1}\right)+\sum_{n\geq t_{N}}\zeta_{n}. \end{array}$$
  - If $t_{N}=N$, then $d(x_{N})=d(x_{t_{N}})$ and we have
    (54) $$d\left(x_{N}\right)-\alpha d\left(x_{N-1}\right)\leq d\left(x_{t_{N}}\right)+\sum_{n\geq t_{N}}\zeta_{n}.$$
  For all n≥1 we define $a_{n}:=d(x_{n})-\alpha d(x_{n-1})$. In both cases it yields
  (55) $$a_{N}\leq d\left(x_{t_{N}}\right)+\sum_{n=t_{N}}^{N}\zeta_{n}\leq d\left(x_{t_{N}}\right)+\sum_{n\geq t_{N}}\zeta_{n}.$$
  Passing in (55) to limit as N→+∞ we obtain that
  (56) $$\underset{n\to +\infty }{lim sup}a_{n}\leq \underset{n\to +\infty }{lim sup}d\left(x_{t_{n}}\right).$$
  Let be u∈S. For all n≥1 we have
  $$d\left(x_{n}\right)=\frac{1}{2}\mathrm{dist}{\left(x_{n},S\right)}^{2}\leq \frac{1}{2}{∥x_{n}-u∥}^{2},$$
  which shows that $(d(x_{n}){)}_{n\geq 0}$ is bounded, as $\lim_{n\to +\infty }∥x_{n}-u∥$ exists. We obtain
  (57) $$\underset{n\to \infty }{lim sup}a_{n}=\underset{n\to \infty }{lim sup}\left[d\left(x_{n}\right)-\alpha d\left(x_{n-1}\right)\right]\geq \left(1-\alpha \right)\underset{n\to \infty }{lim sup}d\left(x_{n}\right)\geq 0.$$
  Further, for all n≥1 we have $(f+g)(x_{t_{n}})<(f+g{)}_{*}$, which gives
  (58) $$\underset{n\to +\infty }{lim sup}(f+g)\left(x_{t_{n}}\right)\leq (f+g{)}_{*}.$$
  This means that the sequence $((f+g)(x_{t_{n}}){)}_{n\geq 0}$ is bounded from above. Consider a subsequence $(x_{t_{k}}{)}_{k\geq 0}$ of $(x_{t_{n}}{)}_{n\geq 0}$ such that
  $$\lim_{k\to +\infty }d\left(x_{t_{k}}\right)=\underset{n\to +\infty }{lim sup}d\left(x_{t_{n}}\right).$$
  From Proposition 3.4 (ii)–(iii) we get that also $(x_{t_{k}}{)}_{k\geq 0}$ and $(h(x_{t_{k}}){)}_{k\geq 0}$ are bounded. Thus, since either *f*+*g* or *h* is inf-compact, there exists a subsequence $(x_{t_{l}}{)}_{l\geq 0}$ of $(x_{t_{k}}{)}_{k\geq 0}$, which converges strongly to an element $\hat{x}$ as l→+∞. According to Proposition 3.4 (ii)–(iii), $\hat{x}$ belongs to arg⁡minh. Furthermore, it holds
  (59) $$\underset{l\to +\infty }{lim inf}(f+g)\left(x_{t_{l}}\right)\geq (f+g)\left(\hat{x}\right)\geq (f+g{)}_{*}.$$
  We deduce from (58) and (59) that
  $$\begin{array}{rl} & (f+g{)}_{*}\leq (f+g)\left(\hat{x}\right)\leq \underset{n\to +\infty }{lim sup}(f+g)\left(x_{t_{l}}\right)\\ & \;\leq \underset{n\to +\infty }{lim sup}(f+g)\left(x_{t_{n}}\right)\leq (f+g{)}_{*}, \end{array}$$
  which gives $\hat{x}\in S$. Thanks to the continuity of *d* we get
  (60) $$\underset{n\to +\infty }{lim sup}d\left(x_{t_{n}}\right)=\lim_{l\to +\infty }d\left(x_{t_{l}}\right)=d\left(\hat{x}\right)=0.$$
  By combining (56), (57) and (60), it yields
  $$0\leq \left(1-\alpha \right)\underset{n\to +\infty }{lim sup}d\left(x_{n}\right)\leq \underset{n\to +\infty }{lim sup}a_{n}\leq \underset{n\to +\infty }{lim sup}d\left(x_{t_{n}}\right)=0,$$
  which implies $\underset{n\to +\infty }{lim sup}d(x_{n})=0$ and thus
  $$\lim_{n\to +\infty }d\left(x_{n}\right)=\underset{n\to +\infty }{lim inf}d\left(x_{n}\right)=\underset{n\to +\infty }{lim sup}d\left(x_{n}\right)=0.$$
2. According to (i) we have $\lim_{n\to \infty }d(x_{n})=0$, thus every weak cluster point of the sequence $(x_{n}{)}_{n\geq 0}$ belongs to S. From Lemma 1.1 it follows that $(x_{n}{)}_{n\geq 0}$ converges weakly to a point in S as n→+∞.
3. Since $\underset{n\to \infty }{lim inf}\lambda_{n}\beta_{n}>0$, from Proposition 3.4(ii) we have that
  $$\lim_{n\to +\infty }∥\nabla h\left(x_{n}\right)∥=\lim_{n\to +\infty }h\left(x_{n}\right)=0.$$
  Since $(x_{n}{)}_{n\geq 0}$ is bounded, there exist $\bar{r}>0$ and $\bar{\kappa }\in R$ such that for all n≥1
  $$∥x_{n}∥\leq \bar{r}\;\mathrm{and}\;h\left(x_{n}\right)\leq \bar{\kappa }.$$
  Thanks to (ii) the sequence $(x_{n}{)}_{n\geq 0}$ converges weakly to an element in S. Therefore, according to Lemma 3.8, it converges strongly to this element in S.
